# Psychometric Properties of the Persian Version of Palliative Care Outcome Scale (POS) in Adult Patients With Cancer

**DOI:** 10.3389/fpsyg.2022.858684

**Published:** 2022-04-28

**Authors:** Masoud Sirati Nir, Maryam Rassouli, Abbas Ebadi, Soolmaz Moosavi, Maryam Pakseresht, Fatemeh Hasan Shiri, Hossein Souri, Morteza Nasiri, Maryam Karami, Armin Fereidouni, Salman Barasteh

**Affiliations:** ^1^Behavioral Sciences Research Center, Nursing Faculty, Baqiyatallah University of Medical Sciences, Tehran, Iran; ^2^Cancer Research Center, Shahid Beheshti University of Medical Sciences, Tehran, Iran; ^3^Behavioral Sciences Research Center, Life Style Institute, Faculty of Nursing, Baqiyatallah University of Medical Sciences, Tehran, Iran; ^4^Department of Medical Surgical Nursing, School of Nursing and Midwifery, Shahid Beheshti University of Medical Sciences, Tehran, Iran; ^5^School of Nursing and Midwifery, Ilam University of Medical Sciences, Ilam, Iran; ^6^Department of Anesthesiology, Faculty of Paramedical, Kashan University of Medical Sciences, Kashan, Iran; ^7^Student Research Committee, Baqiyatallah University of Medical Sciences, Tehran, Iran; ^8^Marine Medicine Research Center, Baqiyatallah University of Medical Sciences, Tehran, Iran; ^9^Department of Rehabilitation Management, University of Social Welfare and Rehabilitation Sciences, Tehran, Iran; ^10^School of Nursing and Midwifery, Shahid Beheshti University of Medical Sciences, Tehran, Iran; ^11^Department of Operating Room Technology, School of Nursing and Midwifery, Shiraz University of Medical Sciences, Shiraz, Iran; ^12^Health Management Research Center, Nursing Faculty, Baqiyatallah University of Medical Sciences, Tehran, Iran

**Keywords:** palliative care, measurement, validity, reliability, psychometrics, scale, symptom, outcome and process assessment

## Abstract

**Background:**

Measuring the outcomes of palliative care plays an important role to improve the quality, efficiency, and availability of these services in patients with cancer. Using valid, reliable, and culturally appropriate tools has a considerable role to measure these outcomes. This study aimed to assess the psychometric properties of the translated version of the Palliative care Outcome Scale (POS).

**Methods:**

This methodological study was conducted in two outpatient clinics related to Shohada Tajrish and Baqiyatallah hospitals in Tehran in 2019–2020. The translation was done using the Forward-Backward approach after gaining permission from the developer. Face validity was tested with 10 patients with cancer through cognitive interviewing, as well as content validity with four experts. Construct validity was performed by (*n* = 203) exploratory factor analysis and confirmation (*N* = 150). To assess the reliability, internal consistency was assessed by using Cronbach's alpha coefficient, and relative stability was assessed using the interclass correlation coefficient (ICC). Furthermore, interpretability and ceiling and floor effects were assessed.

**Results:**

A total of 353 patients with cancer under palliative care were included in the study. Then, three psychological (30%), physical (12.25%), and social factors (12.08%) with a cumulative variance of 54.34% were extracted in exploratory factor analysis. Confirmatory factor analysis showed that the model has a good fit of information. Cronbach's alpha coefficient for scale was 0.719. Furthermore, the ICC was 0.812. The scale was interpretable, and ceiling and floor effects were 0%.

**Conclusion:**

Persian version of the POS was evaluated as a valid and reliable tool. Therefore, it can be used by the clinician to monitor the consequences of palliative care in Iranian cancer patients.

## Introduction

There were an estimated 10 million deaths caused by cancer worldwide in 2020 (Ferlay et al., 2021). In the same year, 131,191 new cases of patients with cancer were diagnosed and 79,136 of them died in Iran (International Agency for Research on Cancer, 2020).

Patients with cancer and their families need physical care, as well as spiritual, psychological, and social support in Iran (Aghaei et al., 2021). Thus, palliative care is used as a response to these needs by improving the quality of life for patients and their families *via* the prevention and relief of pain (Barasteh et al., 2020), reducing the burden of disease, improving satisfaction, and increasing the quality of life of patients (Hugar et al., 2021).

Palliative care is the science and art of improving the quality of life for dealing with a chronic illness over many years (Bakitas et al., 2009). Providing care and supporting patients and their families through the various stages of life-threatening diseases such as cancer are among the goals of palliative care (Aghaei et al., 2021). Receiving palliative care improves the quality of life of patients and their families, but only ~14% of 40 million people needing palliative care each year actually receive it according to WHO (2022).

The establishment and development of palliative care in Iran is one of the goals of the Iranian health system. The first palliative care services in Iran were provided in 2006 at the Comprehensive Cancer Center in Tehran (Rassouli and Sajjadi, 2016). In 2014, the Ministry of Health and Medical Education launched a palliative care working group using various sources and opinions from related fields (Barasteh et al., 2020). At present, palliative care is provided in the form of counseling and comprehensive care in 5–6 centers in Tehran, Isfahan, and Zanjan (Rassouli and Sajjadi, 2016).

Generally, palliative care services in Iran are mostly provided to patients with cancer, although in developed nations, this sort of care is provided to a broad spectrum of patients with terminal conditions, such as heart failure, COPD, and dementia. European Union emphasizes the provision of palliative care at the primary health care level for reasons such as increasing aging populations and the incidence of chronic and debilitating diseases (Heydari, 2018).

At first, providing palliative care faced many challenges in terms of the lack of clear definition, dimensions, and principles according to the cultural context of Iran. The Ministry of Health and Medical Education has helped develop palliative care by helping to form the Iranian Cancer Association and the presence of experts in various fields, as a result of designing and implementing related projects and studies and holding a congress (Barasteh et al., 2021). However, palliative care in Iran encounters a struggle due to weakness in policy-making, insufficient training, shortage of specialists, insurance system concerns, unclear roles and teamwork, medication access issues, and the need for civic support (Barasteh et al., 2020). Also, the development of clinical and research practices is ambiguous due to the lack of standard tools and measurement indicators.

Palliative care professionals must assess the efficacy of the care they offer as the demand for it grows, owing to the inclusion of a broad variety of chronic conditions in patients.

As a result, assessing palliative care outcomes is critical for improving the quality, efficiency, and accessibility of these treatments. Measuring outcomes as a principle in quality assurance and continuous quality improvement (Bausewein et al., 2011), is affected by changes in current and future health status of the patients (Donabedian, 1988; Porter et al., 2016).

In clinical settings, outcome measurement is being used to understand changes in patients' health status or quality of life, to facilitate communication with patients/families and health care team, and to help in clinical decision making and evaluation of the effectiveness of clinical interventions (Gruenewald et al., 2004; Pantaleon, 2019). Palliative outcome care is an essential tool to be used in clinics and research. It has been used for updating clinical practice, monitoring service interventions, and assessing and enhancing the quality of care (Kocatepe et al., 2020).

Patients are the primary source of information on changes in their health status and quality of life, hence the outcomes of health care are linked to their experiences. Examining the effects of palliative care may also aid in achieving the best potential outcomes. Therefore, it is important to use the appropriate tools to measure outcomes in palliative care (Porter et al., 2016).

Thus, various tools including the Memorial Symptom Assessment Scale (MSAS), Edmonton Symptom Assessment Scale (ESAS), and Palliative care Outcome Scale (POS) (Bruera et al., 1991; Aaronson et al., 1993; Roth et al., 1998; Groenvold et al., 2006) are designed to measure the outcomes of palliative care in various dimensions. The POS is a comprehensive tool developed by Hearn and Higginson as a multidimensional benchmark for people with advanced cancer. This scale includes physical and psychological symptoms, spiritual considerations, emotional concerns, and psychological and social needs of the patient and their family (Hearn and Higginson, 1999). One of the important strengths of this tool is its optimal validity and reliability, as well as its design based on a comprehensive care approach. The physical, psychological, social, and spiritual components of the patient's experience are all taken into account while creating this tool (Porter et al., 2016). The tool was translated into various languages, including Spanish (Eisenchlas et al., 2008), Turkish, German (Bausewein et al., 2005), and several other languages.

In Iran, the need for palliative care and its consequences was increasingly considered in recent years. Therefore, by considering the need to study the outcomes of palliative care in the centers which are providing these services in Iran, it is necessary to validate an international tool and provide sufficient information about its psychometric process. Therefore, this study was conducted to translate and assess the psychometric properties of the Persian version of the POS in adult cancer patients.

## Methods

### Study Design

In this methodological study, the POS translated into Persian and assessed its psychometric properties in 2019–2020.

### Study Population/Sampling

This study was performed on patients with cancer who were referred to outpatient clinics Shohada Tajrish and Baqiyatallah and who were in advanced stages of the disease. For this study, 353 patients with cancer were included by the convenience sampling method. Besides, 10 patients participated in the study on face validity. Furthermore, 4 experts were invited for qualitative content validity. Over 18 years old, cancer diagnosis based on physician and patient records, desire in participating in the research, capacity to speak vocally in Persian, and no cognitive or psychiatric issues were the inclusion criteria. Non-cooperation and refusal of the patient or caregiver during the study and incompletion of the scale were considered the exclusion criteria. After obtaining permission, the researcher went to the Baqiyatallah and Shohada Tajrish to start sampling. Statistical Package for the Social Sciences (SPSS) software, version 22.0 was used for data analysis. The maximum error of the first type was considered to be 5%.

### Study Instruments

#### Demographic Information Questionnaire

Researcher-made questionnaire was used to collect the demographic information of the patients, such as age, sex, type of cancer, and painkillers usage history.

#### Palliative Care Outcome Scale (POS)

The POS was developed by Hearn et al. in 1999. It consists of 12 questions, and these questions are related to the patient's physical, mental, emotional, and social states. Except the last question, the answers to all questions are obtained using 0–4 Likert with 0–4 numerical labels. POS scores of each individual question from 1 to 10 can be described in a general score. The overall score is 0–40 so the highest score indicates the maximum disability. Questions 1–8 have 5 options that are rated from zero to four points. Question 9 has three options, and the scores of which are zero, two, and four, respectively. Question 10 has four options, and the points of which are zero, two, four, and zero, respectively. Question 11 completes question 10 and is an open answer. Question 12 of the questionnaire is also related to how to answer the questionnaire, which has 3 points. The measure demonstrated construct validity (Spearman rho = 0.43–0.8). Test/retest reliability was acceptable for seven items. Internal consistency was good (Cronbach's alpha = 0.65) (Hearn and Higginson, 1999).

### Translation Procedure

The translation was done after receiving permission from the developer of the POS. First, the scale was translated from English (main language) to Persian (target language) by two translators (one health specialist and the other general translator). This version was translated back into English by two more translators after analyzing the translations and obtaining a final form from the original Persian version (third and fourth translators). Following the evaluation of the two translations, a copy was retrieved and given to the developer to check the quality of the translation using the International Quality of Life Assessment (IQOLA) translation process (Bullinger et al., 1998).

### Face and Content Validity

Cognitive interviews were conducted to assess the qualitative face validity. In cognitive interviews, the source of error in the tools identified by focusing on the cognitive process of the respondents when filling out the questionnaire (Willis, 2004). Briefly, 10 patients with cancer were interviewed for face validity. Patients were interviewed face-to-face and changes were made to the Persian version of the POS. Furthermore, they were asked to score readability, clarity, item structure, ease of understanding, item complexity, and ambiguous terms, as well as question categorization, ease of replying, language forms, and wording. Furthermore, in order to match the translated version with the original English one and make sure the content can be correctly conveyed, we sent the questionnaire to four experts in Persian Literature to receive their feedback on language forms, diction, and the placement of the words and phrases (qualitative content validity assessment).

### Construct Validity

Patients with cancer receiving palliative care were referred to Shohada Tajrish Center, Baqiyatallah Hospital. Data collection was performed from April 2019 to August 2020. The minimum sample size required for exploratory factor analysis (EFA) is 3–10 participants per item (Plichta, 1965). For EFA, 203 patients with cancer receiving palliative care services were included in the study by available sampling. The principal component analysis (PCA) method was used to extract the factors, and the Promax rotation was used to interpret the factors (Samitsch, 2014).

In order to conduct confirmatory factor analysis (CFA), Munro (2005) recommended 20–30 items per factor (Munro, 2005). Given that the original version of the scale considers 5 dimensions, 100–150 samples were required for CFA. Hence, 150 patients were included in the study for CFA. Indicators of model fit in CFA in three general categories are the following: (1) absolute fit: the root mean square error of approximation (RMSEA), standardized root means square residual (SRMR), Goodness-of-Fit index (GFI), and chi-square; (2) comparative fit: index (CFI), incremental fit index (IFI), relative fit index (RFI), normed fit index (NFI), and Tucker-Lewis index (TLI); (3) affordable fit: parsimony comparative fit index (PCFI), parsimony normal fit index (PNFI), adjusted goodness-of-fit index (AGFI), and Akaike's information criterion (AIC) (Plichta, 1965; Samitsch, 2014).

### Reliability

The correlation among the items referred to internal consistency in a tool that was assessed by Cronbach's alpha coefficient. Furthermore, relative stability was assessed using the interclass correlation coefficient (ICC). The sample size of 28 patients and the interval between two measurements of 1 week were considered.

### Interpretability

To assess the interpretability, the correlation of the total score of scale with gender, age using Pearson Correlation and *t-*test were assessed. Furthermore, the SEM and minimal detectable changes (MDC) were calculated. The following equation was used to calculate the standard error of measurement:


$$\begin{array}{l} \text{SEM}=\text{ SD}\surd 1--\text{ICC} \end{array}$$

Where SD is the standard deviation of the sum values obtained in test and retest phases, while the ICC is the coefficient of repeatability. To calculate the MDC, we used the following equation:


$$\begin{array}{l} \text{MDC}=\text{SEM}\times z\;\surd 2 \end{array}$$

Furthermore, MDC can be calculated as a percentage of the MDC% to determine the actual relative changes after treatment or among repeated measurements over time to further show the relative value of the random error of measurement. To calculate this, the following equation was used:


$$\begin{array}{l} \text{MDC}\%=\left(\text{MDC }÷\text{mean }\right)\times \;100 \end{array}$$

Where “MDC%” is acceptable if it is smaller than 30%, and the excellent MDC% value is assumed to be below 10% (Wu et al., 2011; Sajadi et al., 2020). Percentage of minimum and maximum scores (floor or ceiling effect was considered to be present if >15% of the subjects achieved the lowest or highest possible scores, respectively) (Terwee et al., 2007).

### Data Analysis

We used SPSS software, version 22.0. for EFA and software Linear Structural Relationship (LISREL) version 8.8 for CFA. In all analyses, the significance level was considered *P* < 0.05.

### Ethical Consideration

The present study was conducted in the ethics committee of Baqiyatallah University of Medical Sciences with the ethics code IR.BMSU.REC.1396.134. The researcher described the study approach to all participants and acquired their signed consent after getting the appropriate authorization and coordinating with the relevant authorities. Participants were also told that the study's data would be kept confidential and that they may exit at any moment.

## Results

### Socio-Demographic and Clinical Status

Participants in EFA included 203 patients 95 (46.8%) were men and 108 (53.2%) were women with a mean age of 44.81 ± 14.37. In the CFA, another 150 patients had a mean age of 43.84 years, of which 70 (46.7%) were men and 80 (53.3%) were women. Other details are given in Table 1.

**Table 1:** Rating of exploratory factor analysis (EFA) and confirmatory factor analysis (CFA) based on demographic characteristics.

	**Category**	**EFA**	**CFA**
		***N* (%)**	***N* (%)**
Gender	Male	95 (46.8)	70 (46.7)
	Female	108 (53.2)	80 (53.3)
Age-group (year)	0–30	36 (17.7)	33 (22.00)
	30–50	93 (45.8)	63 (42.00)
	50–70	66 (32.5)	47 (31.3)
	<70	8 (3.9)	7 (4.7)
Type of cancer	Gastrointestinal	76 (37.4)	63 (42.00)
	Blood	37 (18.2)	25 (16.7)
	Uterus and ovary	33 (16.3)	23 (15.3)
	LUNG	16 (7.9)	10 (6.7)
	Others	41 (20.2)	29 (19.3)
Type of pain relief	Non	14 (6.9)	7 (4.7)
	OPOIDS	96 (47.3)	75 (50.00)
	NSAIDS	93 (45.8)	68 (45.3)

### Face and Content Validity

Face validity was confirmed using 10 adult patients with cancer. Moreover, content validity was determined by 4 expert specialists. The items did not change in face and content validity in terms of their simplicity and clarity.

### Construct Validity

#### Exploratory Factor Analysis

The Kaiser-Meyer-Olkin (KMO) test at 0.733 and Bartlett's test were significant (*P* = 0). According to the results, three factors of psychological factors (items 7, 8, 4, and 3), physical factors (items 1, 2, and 9), and social factors (items 6, 10, and 5) were extracted. These three factors accounted for 54.34% of the consequences of palliative care (Table 2).

**Table 2:** EFA of the Persian version of the Palliative care Outcome Scale (POS).

**Factor**	**Item**	**Factor loading**	**variances**	**Eigenvalues**
Factor 1 (emotional dimension)	Q7	0.842	30.007	3.001
	Q8	0.784		
	Q3	0.681		
	Q4	0.631		
Factor 2 (physical dimension)	Q1	0.866	12.256	1.226
	Q2	0.856		
	Q9	0.460		
Factor 3 (social dimension)	Q6	0.751	12.088	1.209
	Q10	0.717		
	Q5	0.441		
Cumulative %	54.351			

#### Confirmatory Factor Analysis

Another sample consisting of 150 patients was selected for CFA. The results of the chi-squared test (x2 = 65.11 and *P* = 0) and other fit incises showed that the three-factor model extracted from EFA has a good fit of the data (RMSEA: 0.072; NFI: 0.88; CFI: 0.93; IFI: 0.93; RFI: 0.82; AGFI: 0.9; PGFI: 0.55; RMR: 0.077; standardized RMR: 0.077).

**Figure 1:**
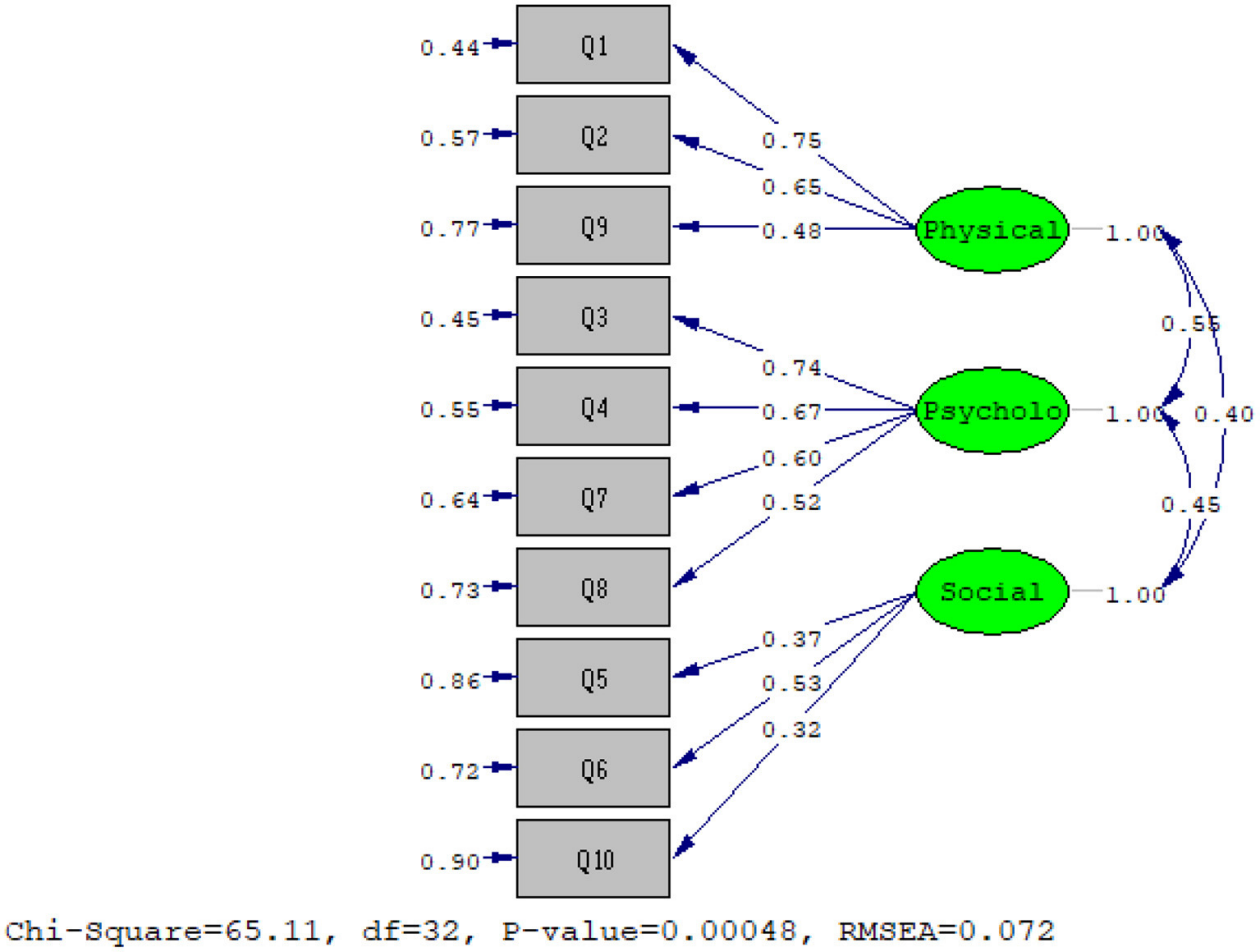
The final structure of the model.

Finally, the results showed that CFA based on the three-factor model extracted from EFA with the obtained data has a good fit (Figure 1).

### Reliability

For reliability, internal consistency with a 95% CI was performed using Cronbach's alpha coefficient (0.719). The total ICC was 0.812. Furthermore, the ICC of the factor was 0.798, the psychological factor was 0.862, and the social factor was 0.77.

### Responsiveness and Interpretability

The result of ANOVA showed that there are no significant differences between the overall POS score and the ages of participants (*P* = 0.739). Moreover, the result of the *t*-test showed that the overall POS score between men and women was not statistically significant (*P* = 0.642). Furthermore, the ceiling and floor effects for the scale were zero, but it was acceptable because it is below 15%. The MDC percentage was calculated at 8.11%. An MDC of <10% was considered excellent. Therefore, MDC was suitable for this study. The SEM was also calculated at 3.81.

## Discussion

The results of the current study deal with the psychometric properties of a 12-item Persian version of the POS in Iran.

Assessing the face and content validity of the scale using the opinions of 10 patients showed that the items are simple and clear. Reviewers judged the measure adequate for tracking palliative care outcomes and authorized coverage beyond the physical issues faced by patients with advanced illness after conducting content validation on the original version of the scale (Hearn and Higginson, 1999). Similar to the results of this study, content validity was confirmed in the Italian version by interviews with patients (Costantini et al., 2016), as well as in the Thai version with CVI report at 0.96 (Pukrittayakamee et al., 2018) and in the Turkish version with CVI report at 0.8–100 (Kocatepe et al., 2020). In addition, the content validity and agreement in the German version of the instrument were evaluated from the perspectives of professional staff and patients in terms of the relevance of the items, forgotten components, and reflection of the patients' true situation. From the perspective of most patients, the tool addressed their real problems, while one-third of them was unsure. Half of the staff were able to communicate with the tool, whereas half were hesitant that the tool could cover patients' real problems. However, the study of Bausewein et al. did not include construct validity due to research limitations (Bausewein et al., 2005).

Confirmatory and exploratory factor analyses of the POS was performed using a sample of 150 and 203 patients with cancer. The fit indices of the Persian version of the POS model based on the main model extracted three psychological, physical, and social factors with a cumulative variance of 54.34%. Goodness-fit indices were confirmed in all three factors. Acceptable values for the RMSEA index (goodness ratios of the mean squared error of the approximation), as well as a 90% CI, should be less than or equal to 0.08. In this study, RMSEA = 0.072 was obtained. The study of Siegert et al. (2010) in the psychometric properties of this scale showed that two factors of “psychological well-being” and “quality of care” were identified, and the three items act separately (Siegert et al., 2010). In the Turkish version of the POS, the compatibility values of the scale (*s* = 69) according to CFA were RMSEA = 0.059, 062, and 0.047 (*P* < 0.05) for the patients, caregivers, and staff, respectively (Kocatepe et al., 2020).

In the research of Harding et al. (2010), the cultural adaptation and psychometric assessment of the POS revealed the tool's excellent qualities, as well as its acceptability and good applicability in clinical settings in Africa. This scale is shorter than similar tools, such as the Missoula-VITAS Quality of Life Index (MVQOLI), and requires less time to complete, which is easy to use in routine clinical evaluations (Harding et al., 2010). The internal stability was acceptable (Cronbach's alpha = 0.719), which is consistent with the results of Eisenchlas et al. (2008).

The ICC of the physical factor was 0.798, the psychological factor was 0.862, and the social factor was 0.77. In the main version of the scale test-retest reliability was acceptable for seven items, and had shown Cronbach's alpha = 0.65 for patients and 0.7 for staff; although they had reported change over time but without statistical significance (Hearn and Higginson, 1999). Internal consistency varies in different studies. The internal consistency in the Thai version scale was 0.9 (Pukrittayakamee et al., 2018), while the Argentine version scale was acceptable at Cronbach's alpha = 0.68–0.69 and 0.66–0.73 for patient and staff ratings, respectively, and test-retest reliability showed very high agreement for every item (>0.8) (Eisenchlas et al., 2008). Although Cronbach's alpha of 0.6 in the African POS had shown moderate internal stability of the scale, test-retest has shown high intra-class correlation coefficients for all items (0.78–0.89) (Harding et al., 2010). Furthermore, Cronbach's alpha of 0.64 in the Turkish POS had shown moderate internal stability of the scale (Kocatepe et al., 2020).

According to the psychometric properties of the Persian version of the POS, the construct validity can be concluded that the mentioned tool in three factors, namely emotional, physical, and social, covers the outcomes of palliative care in patients with cancer. Based on the research of Kocatepe et al. (2020), the Turkish POS is also a valid and reliable tool that can be used with patients, caregivers, and staff members in three dimensions for the evaluation of physical and psychological symptoms, including spiritual, practical, and emotional concerns, as well as psychosocial needs (Kocatepe et al., 2020). Pukrittayakamee et al. (2018) showed that this tool is valid and reliable for use in primary research and clinical setting (Pukrittayakamee et al., 2018).

In this study, the MDC was calculated at 8.11%. A minimum percentage of detectable change of <10% was considered excellent. Therefore, MDC was suitable for this study. The SEM was also calculated at 3.81. These indicators suggested the desirable characteristics of the scale. However, these features have not been studied in similar studies.

Finally, it can be concluded that this tool is suitable for use in clinical settings to assess the symptoms and concerns of the patients and monitor changes in them over time. The tool is concise and takes <10 min to complete. This tool is widely evaluated and is available in various language versions. This tool can be used in a wide range of diseases and different clinical settings such as hospitals, nursing homes, and hospices (Van Vliet et al., 2015).

## Conclusion

The results of this study showed that the POS tool has a favorable face, content, and structural validity (including EFA and CFA). The reliability of the tool was also reported as desirable. Therefore, due to the need of the Iranian community for palliative care, this tool is suggested to be widely used in clinical, educational, and research in Iran.

### Limitation

One of our limitations was the absence of psychometric methods to assess the study's criterion validity. Iranian scholars may utilize these tools to investigate additional validation approaches such as criterion and concurrent validity after psychometric examination of numerous acceptable measures in the area of palliative care.

## Data Availability Statement

The original contributions presented in the study are included in the article/supplementary material, further inquiries can be directed to the corresponding author.

## Author Contributions

MS, SB, AE, and MR: conceptualization, methodology, draft preparation, and supervision. SM, MP, FH, HS, and AF: writing-reviewing and editing, data collection, and draft preparation. MN, MK, AE, and SB: visualization, investigation, software, data collection, and draft preparation. All authors contributed to the article and approved the submitted version.

## Conflict of Interest

The authors declare that the research was conducted in the absence of any commercial or financial relationships that could be construed as a potential conflict of interest.

## Publisher's Note

All claims expressed in this article are solely those of the authors and do not necessarily represent those of their affiliated organizations, or those of the publisher, the editors and the reviewers. Any product that may be evaluated in this article, or claim that may be made by its manufacturer, is not guaranteed or endorsed by the publisher.
